# Non-contrast agent based small vessel imaging of human thyroid using motion corrected power Doppler imaging

**DOI:** 10.1038/s41598-018-33602-9

**Published:** 2018-10-17

**Authors:** Rohit Nayak, Viksit Kumar, Jeremy Webb, Adriana Gregory, Mostafa Fatemi, Azra Alizad

**Affiliations:** 1 Department of Radiology, Mayo Clinic College of Medicine and Science, Rochester, Minnesota 55905 United States; 2 Department of Physiology and Biomedical Engineering, Mayo Clinic College of Medicine and Science, Rochester, Minnesota 55905 United States

## Abstract

Singular value based spatiotemporal clutter filtering (SVD-STF) can significantly improve the sensitivity of blood flow imaging in small vessels without using contrast agents. However, despite effective clutter filtering, large physiological motion in thyroid imaging can impact coherent integration of the Doppler signal and degrade the visualization of the underlying vasculature. In this study, we hypothesize that motion correction of the clutter filtered Doppler ensemble, prior to the power Doppler estimation, can considerably improve the visualization of smalls vessels in suspicious thyroid nodules. We corroborated this hypothesis by conducting *in vivo* experiments on 10 female patients in the age group 44–82 yrs, with at least one thyroid nodule suspicious of malignancy, with recommendation for fine needle aspiration biopsy. Ultrasound images were acquired using a clinical ultrasound scanner, implemented with compounded plane wave imaging. Axial and lateral displacements associated with the thyroid nodules were estimated using 2D normalized cross-correlation. Subsequently, the tissue clutter associated with the Doppler ensemble was suppressed using SVD-STF. Motion correction of the clutter-filtered Doppler ensemble was achieved using a spline based sub-pixel interpolation. The results demonstrated that power Doppler images of thyroid nodules were noticeably degraded due to large physiological motion of the pulsating carotid artery in the proximity. The resultant power Doppler images were corrupted with signal distortion, motion blurring and occurrence of artificial shadow vessels and displayed visibly low signal-to-background contrast. In contrast, the power Doppler images obtained from the motion corrected ultrasound data addressed the issue and considerabley improved the visualization of blood flow. The signal-to-noise ratio and the contrast-to-noise ratio increased by up to 15.2 dB and 12.1 dB, respectively. Across the ten subjects, the highest improvement was observed for the nodule with the largest motion. These preliminary results show the ability of using motion correction to improve the visualization of small vessel blood flow in thyroid, without using any contrast agents. The results of this feasibility study were encouraging, and warrant further development and more *in vivo* validation in moving tissues and organs.

## Introduction

In United States, there are over 50,000 new cases of thyroid cancer every year, with a likelihood of 3:1 for females to males^1^. However, 60–80% of thyroid cancer diagnosis by fine needle aspiration (FNA) biopsies result in benign findings, which unnecessarily causes significant physical and financial burden to the patients and the health system^2,3^. Ultrasound sonography can serve as an ideal candidate for non-invasive diagnosis of suspicious thyroid nodules. However, its low specificity leads to a large number of FNAs.^4^.

Several researches have demonstrated the potential of using non-invasive ultrasound based Doppler blood flow imaging to detect thyroid malignancy based on the assessment of intranodular and peripheral vascularity^5–14^. However, a major challenge with Doppler imaging of thyroid nodules is that large tissue motion due to its proximity to the pulsating carotid artery can impact the performance of tissue clutter suppression and PD integration, which are important factors for blood flow visualization. Recent studies have demonstrated that spatio-temporal based clutter rejection can substantially improve the sensitivity of non-invasive small vessel blood flow imaging^15,16^. This was achieved by singular value decomposition (SVD) based spatio-temporal clutter filtering of ultrafast Doppler ensemble. Specifically, in ultrafast Doppler ensembles, tissue components are primarily low rank due to its higher temporal and spatial coherence compared to the incoherent flow of blood. Accordingly, in the presence of motion, a singular value rank based approach can be more effective in suppressing tissue clutter than conventionally used frequency based filters^15,16^.

**Figure 1:**
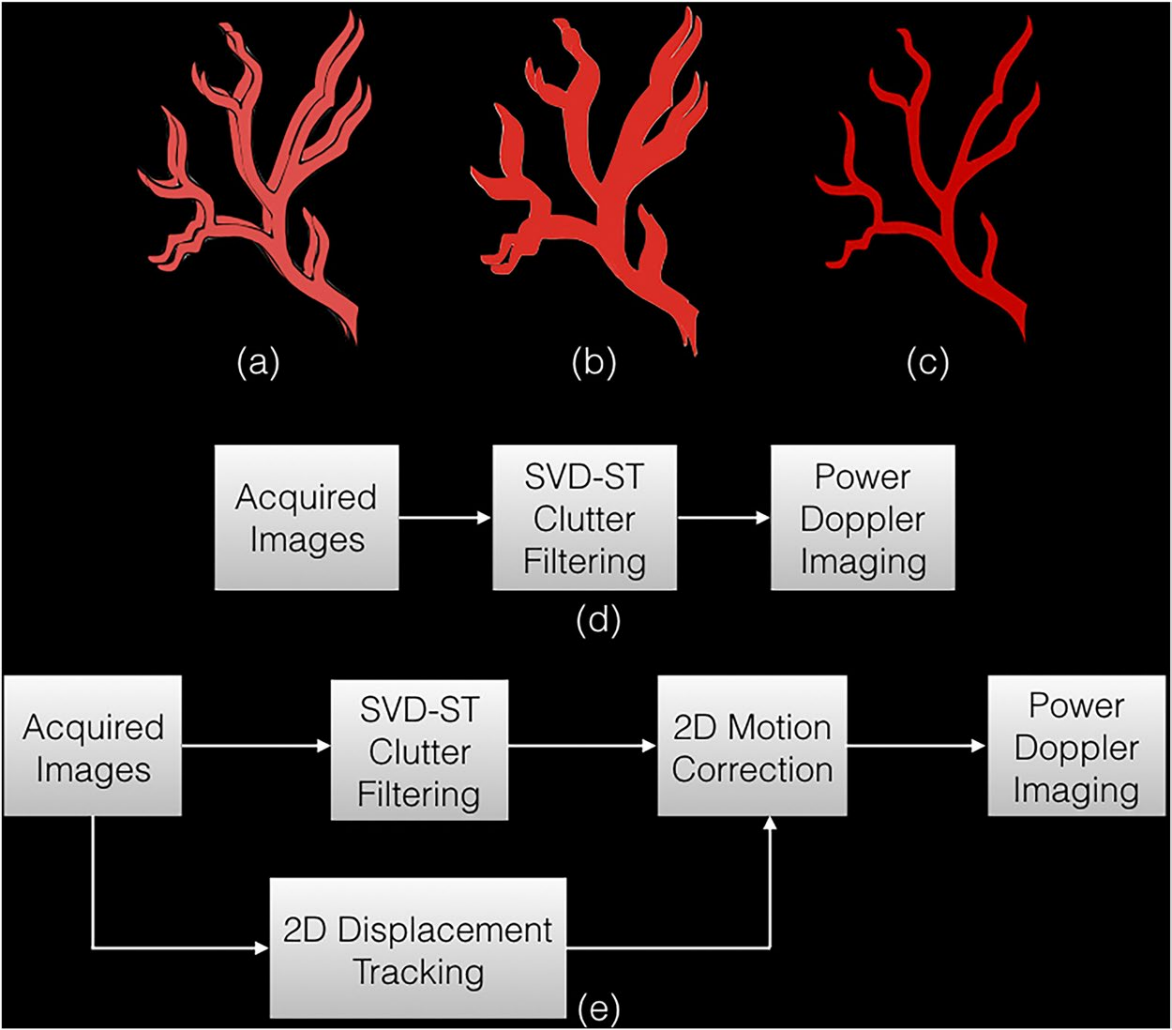
An illustration to display the impact of motion on blood flow visualization. (**a**) displays the clutter filtered blood flow signal in two example Doppler frames, spatially mis-registered due to motion. On estimating the power Doppler image using Eqn. (2), the final image is blurred with appearance of shadow vessels. (**c**) displays the final image expected upon motion correction, where the Doppler signals coherently integrate to produce a more stronger signal with reduced motion artifacts. Specifically, (**b**) and (**c**) are obtained using the approach outlined in (**d**) and (**e**), respectively.

However, even if tissue clutter is completely suppressed in the presence of motion, estimation of the cumulated power Doppler signal from the motion affected clutter filtered data can be challenging due to miss-registration of the ultrasound frames in the Doppler ensemble. This can reduce the sensitivity of detecting small vessels. Specifically, PD signal is computed from coherent integration of the clutter filtered signal. However, in the presence of motion, which is unavoidable in thyroid imaging, signal incoherency due to spatial miss-registration of the ultrasound frames can considerably degrade the image quality (Fig. 1a–c). Further, imaging of blood flow in small vessels – typically characterized by low intensity back-scatter signals, can be particularly unreliable, since motion can easily corrupt its contribution due to incoherent integration. The impact of motion on small vessel imaging can typically be mitigated by using high frame-rate imaging, however, the need for angular compounding of the ultrasound frames to increase the signal-to-noise ratio of plane wave imaging can reduce the frame-rate by up to an order of magnitude^15–17^, which may lead to increased motion between successive frames. Additionally, imaging of large or deep seated thyroid nodules can further penalizing the imaging frame-rate due to increased scan depth. This loss of blood flow visualization can potentially be improved by injecting micro-bubble based contrast agents in the blood stream^18^. However, it is associated with increased complexity, cost and invasiveness.

In this study, we investigated the feasibility of using motion corrected Doppler ensemble for non-invasive *in vivo* small vessel imaging of human thyroid (Fig. 1e), and compared its performance with the previous approach (Fig. 1d). The hypothesis of this paper is that estimation of power Doppler image from motion corrected Doppler ensemble can enable coherent integration of blood signal, and thus improve the visualization and detection of small vessels.

The proposed hypothesis of the study is based on the outcome of recent ultrasound contrast agent based micro-vasculature imaging studies^19,20^ that used a similar technique for improving the blood flow visualization. The efficacy of their technique was demonstrated on a rat model, and the results showed an improvement in the definition of the renal micro-vessels, which were otherwise blurred due to motion. In non-contrast agent based blood flow imaging – the focus of this study, the signal from blood flow in small vessels can be up to 60–80 dB lower than that of tissue^21–23^. Therefore, the impact of motion and motion correction can be expected to be higher and more significant.

In this paper, we tested this hypothesis using normalized cross-correlation (NCC) based speckle tracking, which has been the gold standard for high quality motion estimation in ultrasound imaging^24^, and has been widely used for blood flow imaging^25^, elastographic imaging^26–28^, temperature imaging^29^, phase-aberration correction^30^.

We used a 2D NCC based speckle tracking technique to estimate tissue displacements, which were subsequently used for motion correction. We demonstrated the efficacy of the proposed technique by conducting *in vivo* imaging of thyroid glands using a clinical ultrasound scanner implemented with compounded plane wave imaging.

In the following sections, first, the displacement estimation and the motion correction techniques are described, followed by detailed analysis and comparison of results obtained from the patients using the previous (Fig. 1c) and the proposed (Fig. 1d) techniques.

## Results

**Figure 2:**
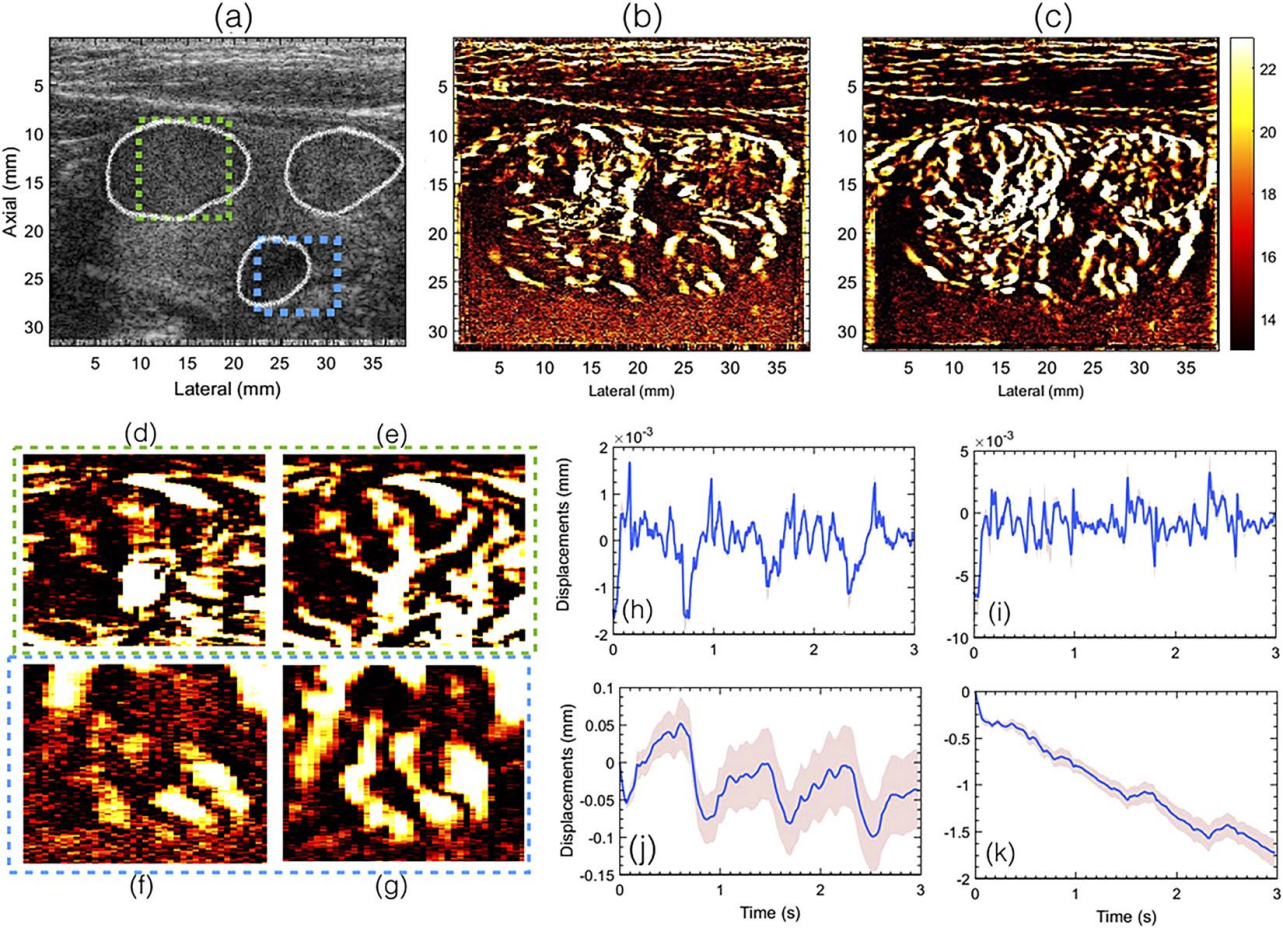
Displays the plane wave B-mode sonogram (**a**) and the corresponding PD images without (**b**) and with motion (**c**) correction. The outline of the thyroid nodules are indicated in white in the B-mode sonogram (**a**). Figures (**d**,**f**) show zoomed insets obtained from (**b**). Figures (**e**,**g**) show zoomed insets obtained from (**c**). The green and blue outline corresponds to images in (**d**,**f**) and (**e**,**g**), respectively. Figures (**h**,**i**) and (**j**,**k**) visualize the axial and lateral displacements, respectively. Further, (**h**,**i**) displays the displacements associated with every consecutive frame, and (**j**,**k**) displays the total accumulated displacements, with reference to the first frame in the ensemble. The continuous error-band (red) displays ±1 standard deviation from the mean. For the sake of clarity in visualizing the small blood vessel structures, the green and the blue ROIs were indicated in the B-mode sonogram.

**Figure 3:**
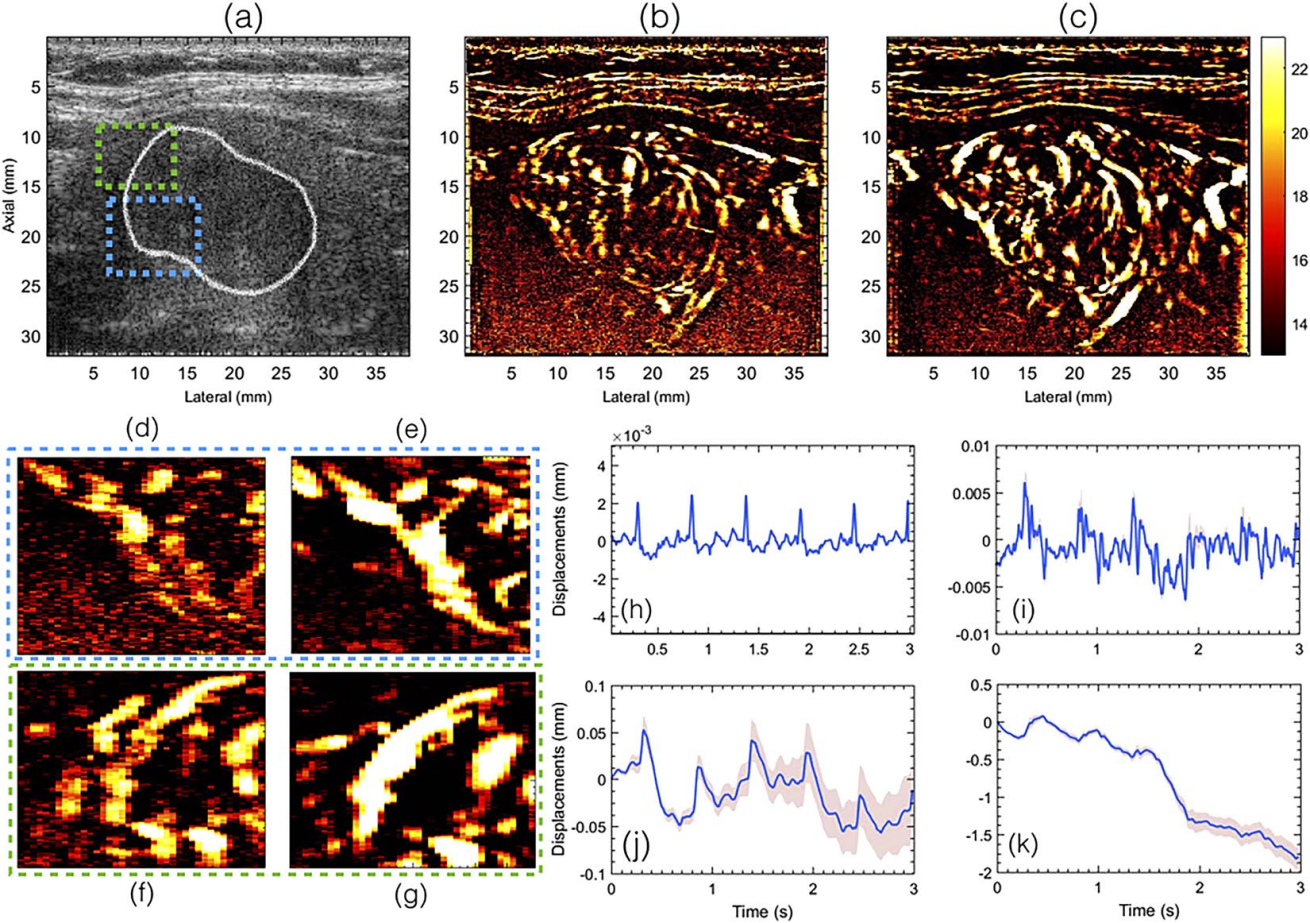
Displays the plane wave B-mode sonogram (**a**) and the corresponding PD images without (**b**) and with motion (**c**) correction. The outline of the thyroid nodule is indicated in white in the B-mode sonogram (**a**). Figures (**d**,**f**) show zoomed insets obtained from (**b**). Figures (**e**,**g**) show zoomed insets obtained from (**c**). The green and blue outline corresponds to images in (**e**,**g**) and (**d**,**f**), respectively. Figures (**h**,**i**) and (**j**,**k**) visualize the axial and lateral displacements, respectively. Further, (**h**,**i**) displays the displacements associated with every consecutive frame, and (**j**,**k**) displays the total accumulated displacements, with reference to the first frame in the ensemble. The continuous error-band (red) displays ±1 standard deviation from the mean.

Representative examples of the *in vivo* thyroid sonograms obtained from patients 1 and 2 are displayed in Figs 2(a) and 3(a), respectively. The boundaries of the nodule in the thyroid sonogram are demarcated in white. The corresponding power Doppler images obtained without (Fig. 1d) and with (Fig. 1e) motion correction are displayed in (b) and (c), respectively. Further, the zoomed PD insets from the two ROIs indicated in (a) are displayed in (d–g). Motion correction for (c) was applied post clutter filtering, as outlined in Fig. 1e. Subplots (h–k) displays the mean axial and lateral displacements associated with the thyroid nodules, computed using 2D normalized cross-correlation based speckle tracking. The inter-frame displacements (h,i) were computed between each consecutive ultrasound frame, prior to clutter filtering, and the corresponding accumulated displacements are reported in (j,k). The mean axial and lateral displacements reported in (h,i) were averaged over the entire thyroid nodule that was outlined by an expert sonographer. The corresponding ±1 standard deviation band were reported in red, which were observed to be negligible in (h,i) since the variation of displacements in the nodule were small, but increased upon accumulation of displacements (j,k) due to increase in number of frames. The pulsating motion of the carotid artery could be observed in the axial and lateral displacement plots. However, it was relatively more distinct in the displacements estimated from patient 2 than patient 1, and this may depend on factors such as amplitude of the carotid pulsation, size and stiffness of the nodule and its distance the carotid artery^12,31^. Further, a steady amount of translational motion was also observed that could be due to the slipping of the nodule or the probe during image acquisition.

The results show that the power Doppler images estimated from the motion corrected ensemble displayed a noticeable improvement in image quality. These observations were consistent with previously reported studies involving contrast agent micro-bubbles for imaging renal vasculature in a rat model^19^. Motion induced signal distortion, blurring and appearance of shadow vessel that corrupted the PD images were considerably reduced upon motion correction.

**Figure 4:**
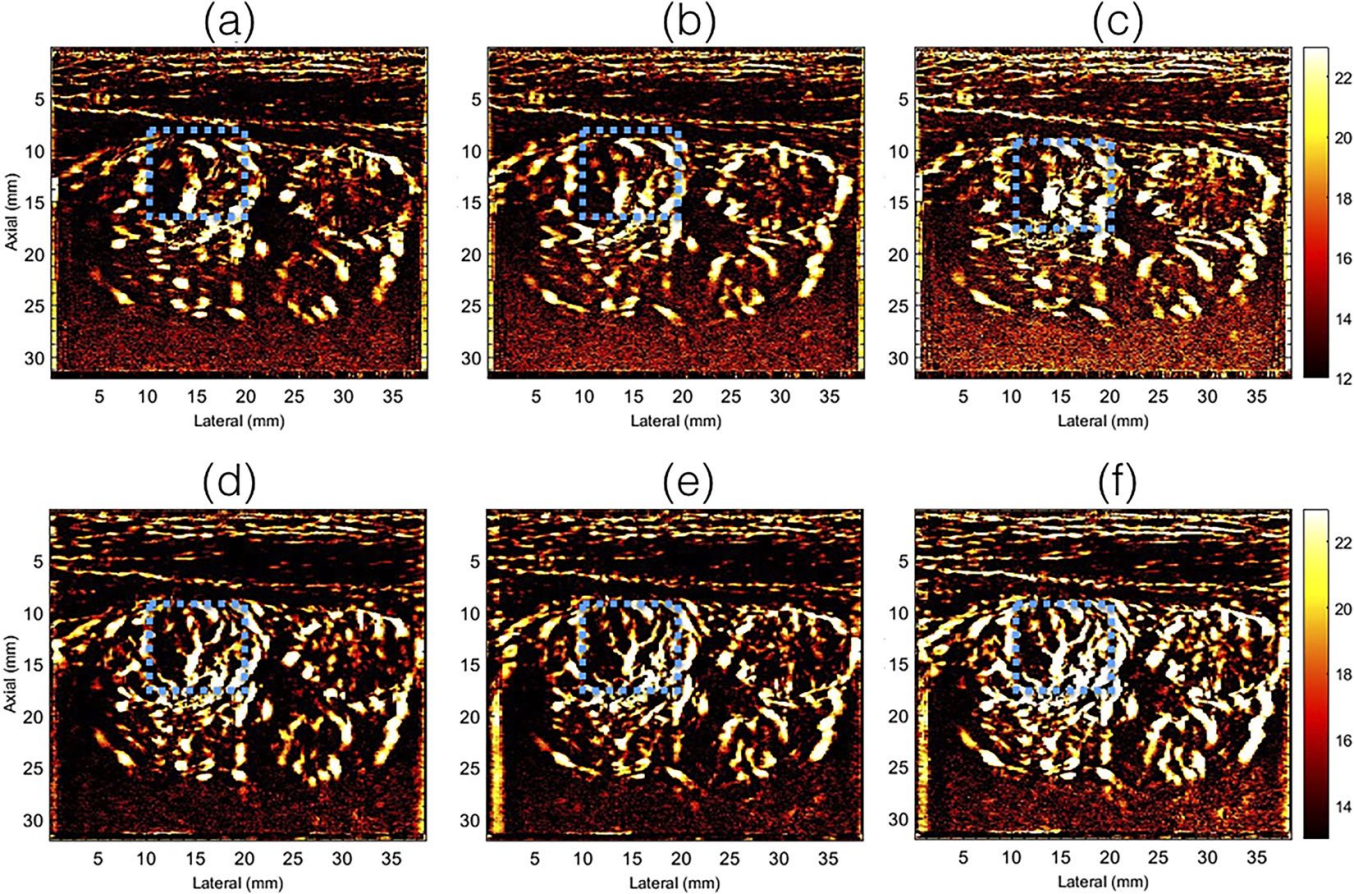
Displays the half- and full-ensemble PD images obtained using clutter filtered Doppler ensemble, without (**a**–**c**) and with (**d**–**f**) motion correction. The half ensemble PD images are displayed in (**a**,**b**,**d**,**e**), and the corresponding full-ensemble PD images were displayed in (**c**,**f**). The blue ROI indicates a region for closer observation and comparison.

**Figure 5:**
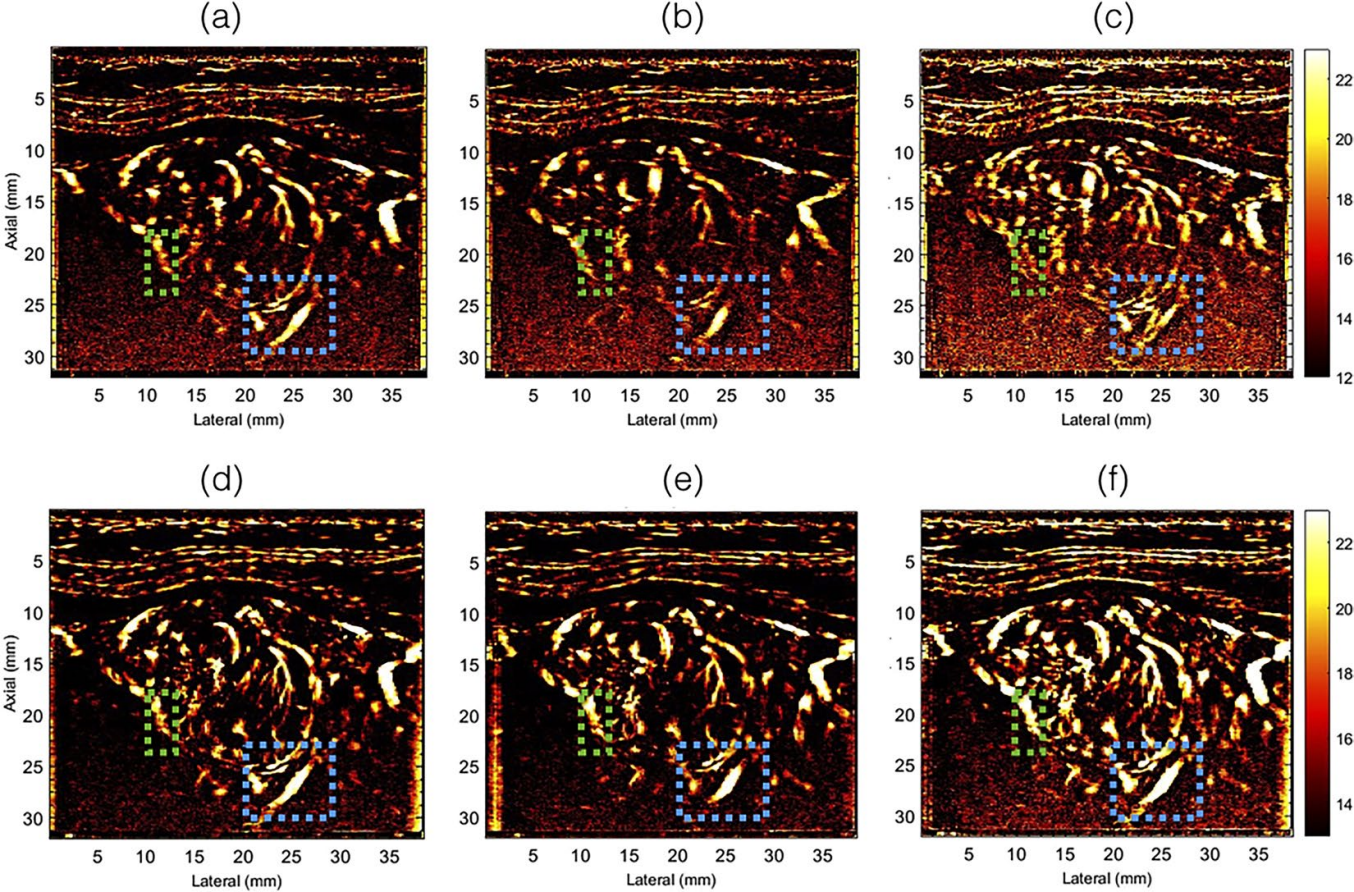
Displays the half- and full-ensemble PD images obtained using clutter filtered Doppler ensemble, without (**a**–**c**) and with (**d**–**f**) motion correction. The half ensemble PD images are displayed in (**a**,**b**,**d**,**e**), and the corresponding full-ensemble PD images were displayed in (**c**,**f**). The blue and green ROI indicates two regions for closer observation and comparison.

Figures 4 and 5 display the half and full ensemble power Doppler images obtained from patients 1 and 2, respectively, without (a–c) and with (d–f) motion correction. The PD images displayed in columns 1 and 2 were computed from the first (0–1.5 secs) and second (1.5–3 secs) half of the Doppler ensemble, respectively. Column 3 (c,f) displays the corresponding PD images obtained using the full ensemble.

The half ensemble data incurred relatively less motion than the corresponding full ensemble (Fig. 2(h–k)). Therefore, fewer motion artifacts were expected in the PD images, however, at the cost of signal strength, which was observed in (a–c). However, there were two key observations from these results. First, in the absence of motion correction, the vessel features observed in the half-ensemble PD images were similar to those obtained post-motion correction. Second, the vessel features observed in the motion corrected half ensemble PD images (d,e) were relatively more consistent with the full-ensemble PD image (f), which constructively integrated to produce an ever stronger signal in (f) than (d,e). This was not the case in (a–c). Specifically, in the absence of motion correction, the full-ensemble PD image (c) displayed more motion artifacts that those observed in the half ensemble images (a,b). The blue and the green ROIs in Figs 4 and 5 indicate some of the specific regions for closer observation across subplots (a–f).

**Figure 6:**
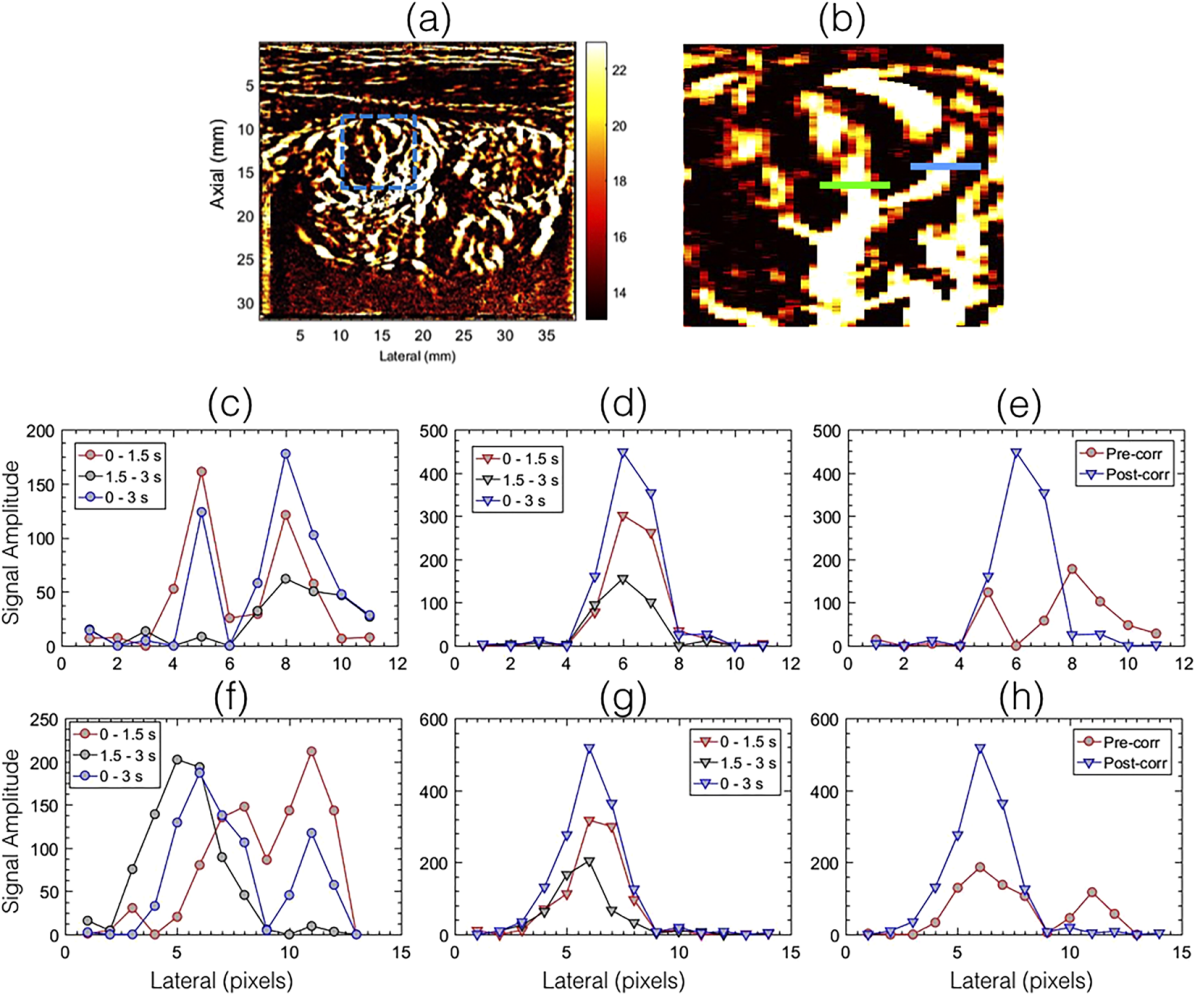
Displays the spatial amplitude profile obtained from the half- and full -ensemble PD images displayed in Fig. 4. Signal amplitude profiles (**c**–**e**) and (**f**–**h**) corresponds to the blue and green line segments in the zoomed inset (**b**) of the PD image in (**a**), respectively. In the absence of motion correction (**c**,**f**), coherent integration of the Doppler data was not feasible, which led to poor signal reconstruction. In the motion corrected data, the half- ensemble PD intensity profiles (**d**,**g**) were spatially registered, which produce a stronger peak (**e**,**h**).

Figure 6 displays representative signal amplitude profiles obtained from the PD images displayed in Fig. 4. The blue and green line segments in the zoomed inset (b), obtained from the PD image (a), corresponds to the data displayed in (c–e) and (f–h), respectively. The red, black and blue plots in (c, f) correspond to the pre-corrected PD profiles obtained from Fig. 4(a–c), respectively. Similarly, the red, black and blue plots in (d,g) corresponds to the post-corrected PD images, displayed in Fig. 4(c–e), respectively. These results reveal the spatial mis-registration in the Doppler signal, in the absence of motion correction, which led to motion artifacts in the PD images. In comparison, the results obtained using the proposed technique revealed that the motion corrected PD signals were well aligned, and coherently integrated, which improved the signal strength and minimized motion artifacts in the power Doppler images.

**Figure 7:**
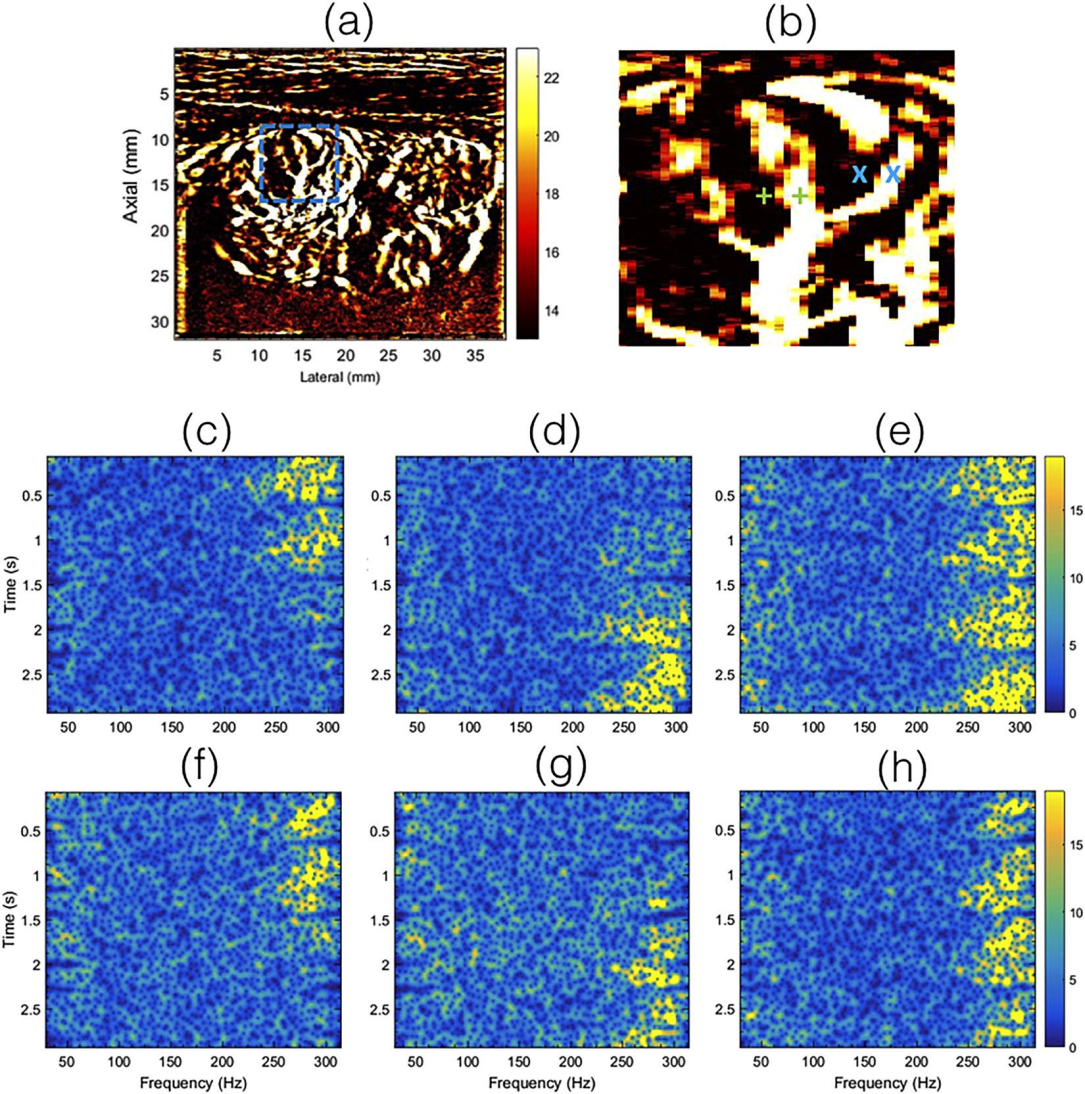
Displays the time-frequency spectrogram associated with the clutter filtered Doppler ensemble, prior to PD computation. Each time-frequency spectrum was calculate at a single-pixel spatial location, indicated in zoomed inset (**b**), obtained from the PD image (**a**). Specifically, figures (**c**–**e**) and (**f**–**h**) corresponds to the green ‘+’ and blue ‘x’ in (**b**), respectively. The time-frequency spectrums displayed in (**c**,**f**) and (**d**,**g**) were obtained without motion correction. Data displayed in (**c**,**d**) corresponded to the locations indicated by the left and right green ‘+’ in (**b**), respectively, prior to motion correction. Similarly, the data displayed in (**f**,**g**) corresponded to the locations indicated by the left and right blue ‘x’ in (**b**), respectively, prior to motion correction. Each of the green ‘+’ and blue ‘x’ pixel pairs were separated by 4 pixels. The time-frequency spectrums for the motion corrected data were displayed in (**e**,**h**), obtained from the location indicated by the right ‘+’, ‘x’ markers in (**b**), respectively.

Figure 7 displays the time-frequency spectrum associated with the clutter filtered Doppler ensemble, obtained from the individual pixel locations indicated by green ‘+’ (c–e) and blue ‘x’ (f–h) markers in the zoomed inset (b), corresponding to the PD image in (a). Each pixel pairs indicated in green and blue were separated by 4 pixels. Columns 1 (c,f) and 2 (f,g) show the spectrogram associated with uncorrected Doppler ensemble, at two adjacent locations, separated by 4 pixels laterally. The third column (e,h) shows the corresponding spectrogram obtained from the motion corrected Doppler ensemble, corresponding to ‘+’ and ‘x’ pixel locations on the right side.

The time frequency spectrum associated with blood flow was relatively narrow-band and the inherent pulsation in the blood flow was visible, similar to those typically observed in pulse wave spectral Doppler imaging^32,33^. For small vessel imaging of thyroid, in the absence of motion correction, the time-frequency spectrum was discontinuous, and was observably split over multiple adjacent location. In comparison, the motion corrected time-frequency spectrum displayed a continuous signal in time, and was located at a single vessel location, which was consistent with observations in Figs 4–6. The quality of the spectrograms was not as good as those typically obtained from focused ultrasound beams, however, it was observed to be comparable to those reported previously using compounded plane wave imaging^33^. Typically, spectral Doppler imaging is performed for a specific location at high pulse repetition frequency; with the current approach, the maximum frequency was limited to 308 Hz. Further, in spectral Doppler, the spectrum is estimated from a gated region, however, in this analysis the results were obtained from an individual pixel to specifically analyze the impact of motion and correction on Doppler spectrum^34^.

**Figure 8:**
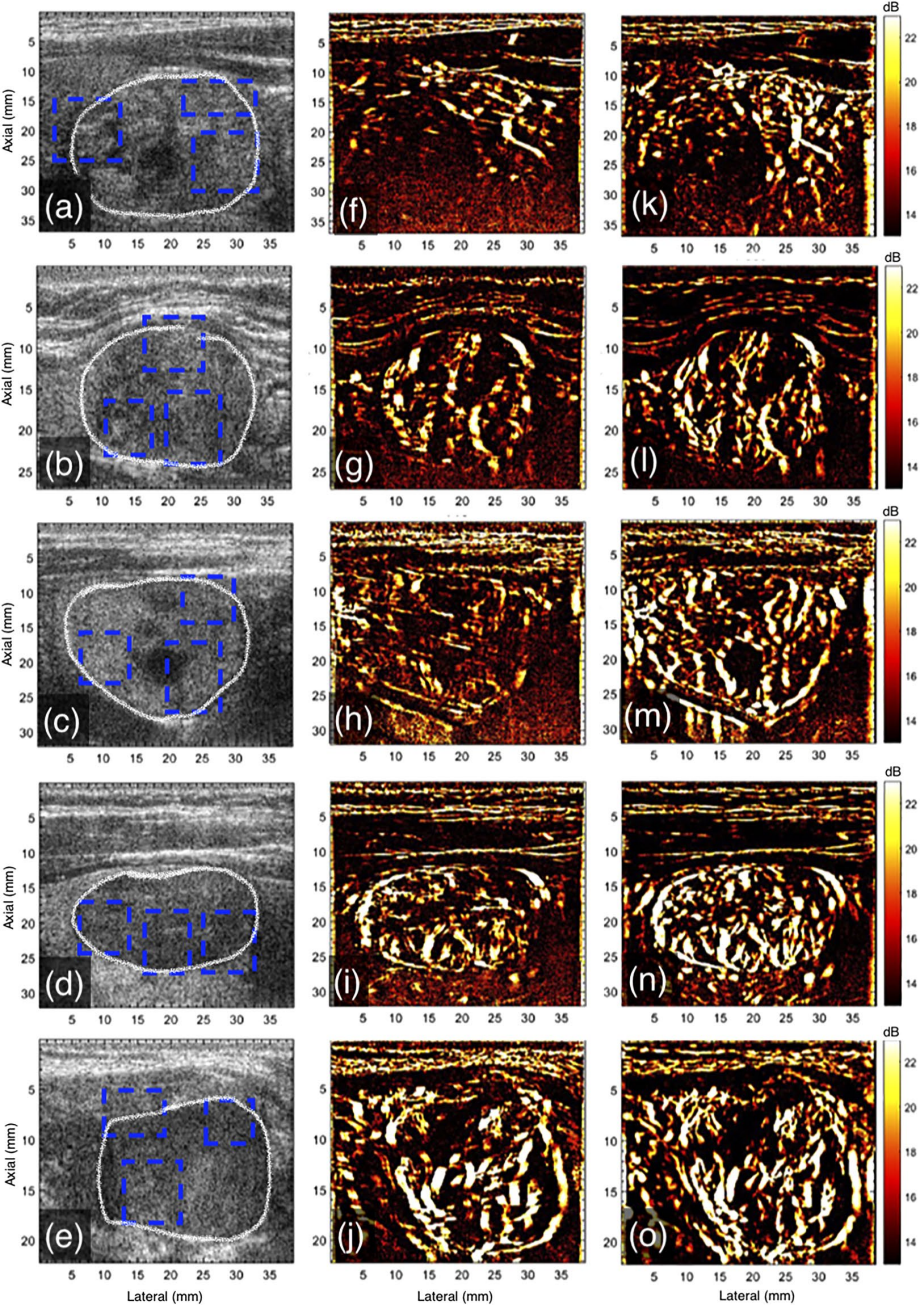
Displays the plane wave B-mode sonograms (**a**–**e**) and the corresponding PD images without (**f**–**j**) and with (**k**–**o**) motion correction, for patients 3–7, respectively. The outline of the thyroid nodules is indicated in white in the B-mode sonogram (**a**–**e**). For the sake of clarity in visualizing the small blood vessel structures in the PD images, the blue ROIs that were used for quantitative analysis in Fig. 9 were indicated in the B-mode sonograms.

**Figure 9:**
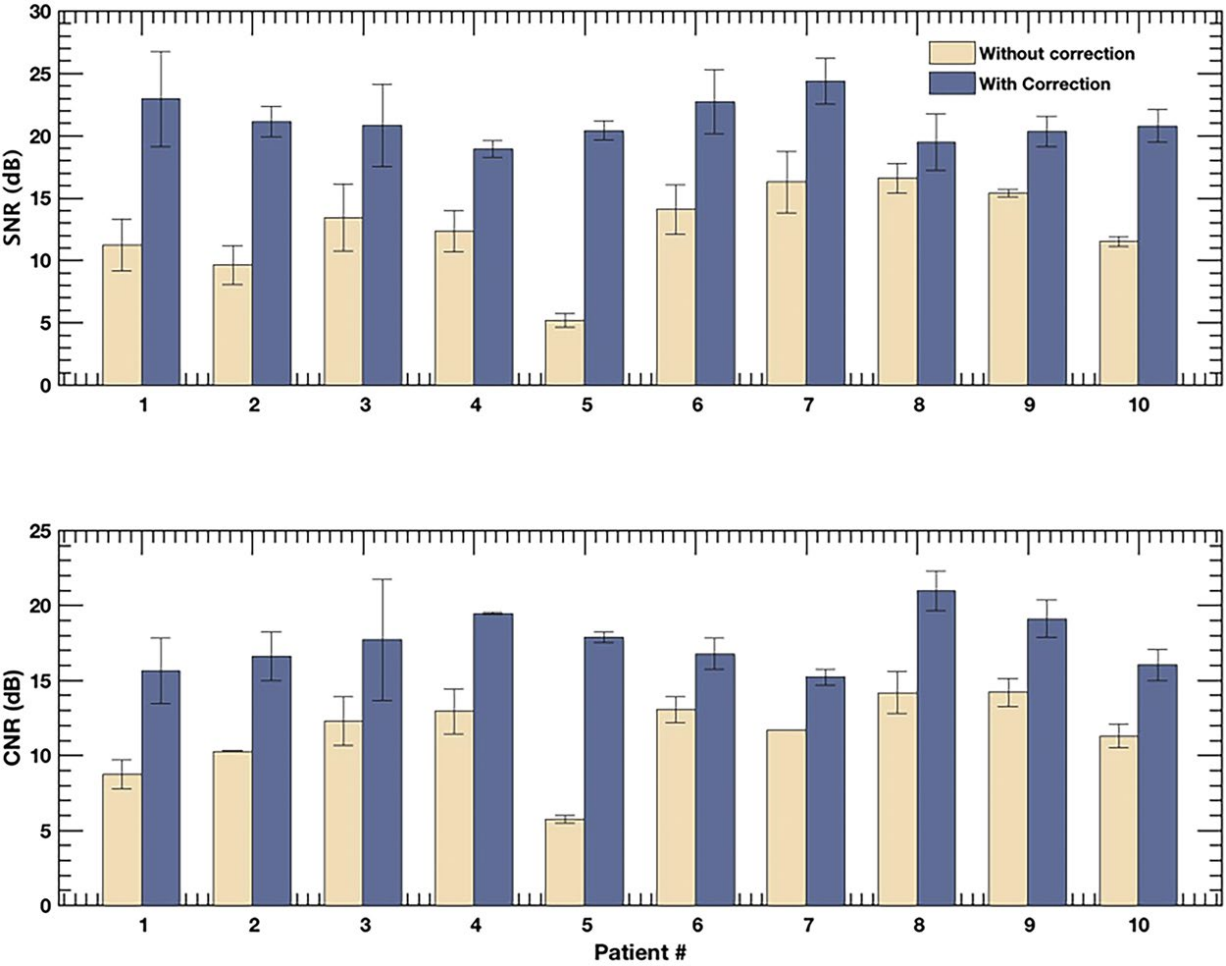
Displays barplot of SNR (**a**) and CNR (**b**) associated with the PD images of the 10 patients, with and without motion correction. The errorbar corresponds to the ±1 standard deviation. The quantitative metrics were computed in the blue ROIs indicated in Figs 2, 3 and 8.

The montage in Fig. 8 displays representative examples of *in vivo* plane wave sonograms (a–e) and the corresponding PD images, without (f–j) and with motion (k–o) correction, for patients 3–7. The improvement in the quality of the power Doppler images upon motion correction was noticeable for all cases. The most significant improvement was observed in PD image obtained from patient 5, which also incurred the largest motion (Fig. 9). The PD image obtained prior to motion correction was considerably degraded due to lack of coherent integration of the Doppler signal.

**Figure 10:**
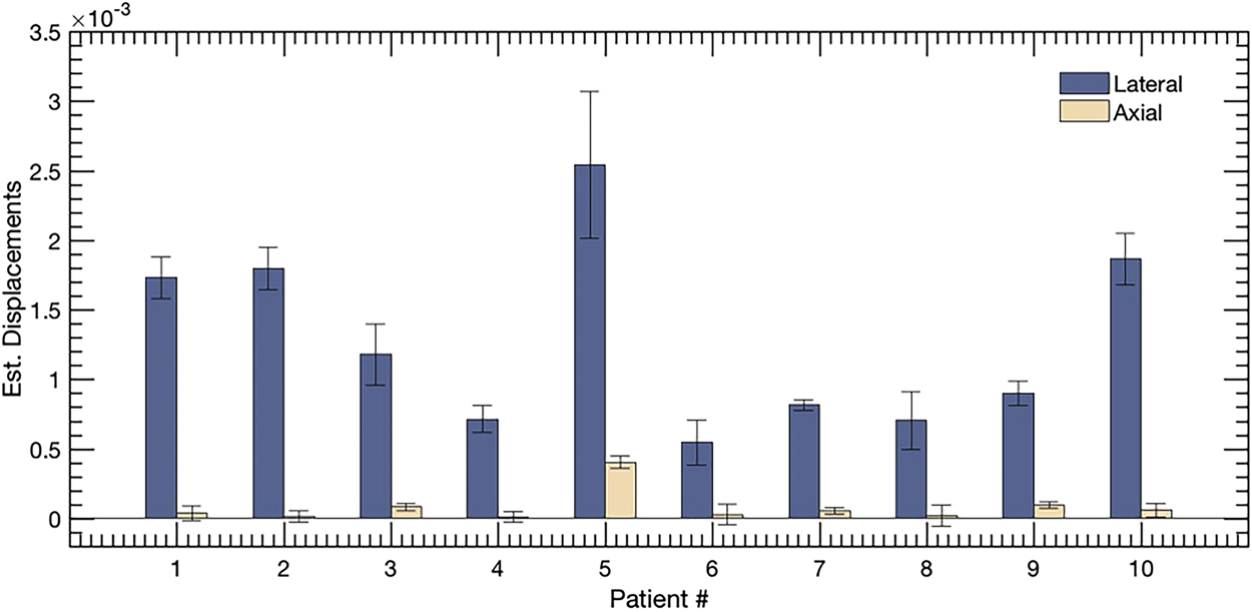
Displays the barplot of cumulated lateral and axial displacement estimates of the thyroid lesion obtained from 10 subjects. The displacements were computed in lesion ROI indicated in the sonograms in Figs 2, 3 and 8.

In Fig. 9 are bar plots of SNR (a) and CNR (b) of the power Doppler images computed with and without motion correction. The quantitative metrics were computed from the rectangular ROI highlighted in Figs 2, 3 and 8, using eqs 3 and 4, respectively. Specifically, CNR and SNR were computed in each of the three ROIs and averaged. This information was displayed in barplots with errorbars in Fig. 9, represented by mean and ±1 standard deviation, respectively. The improvement in the SNR and CNR varied from 2.9 dB to 15.2 dB and 3.7 dB to 12.1 dB, respectively, across the patients considered in this study. The highest improvement in SNR and CNR was observed for patient # 5, which also recorded the largest tissue displacement (Fig. 10).

In Fig. 10 are bar plots of the mean axial and lateral displacements. The lateral displacements were predominantly higher than axial, which was expected from the anatomical positioning of the thyroid with respect to the pulsating carotid artery. The measured displacements were observed to vary across patients, which could be due to multiple factors, such as the size, stiffness, location and mobility of the nodule and the magnitude of the carotid pulsation.

**Figure 11:**
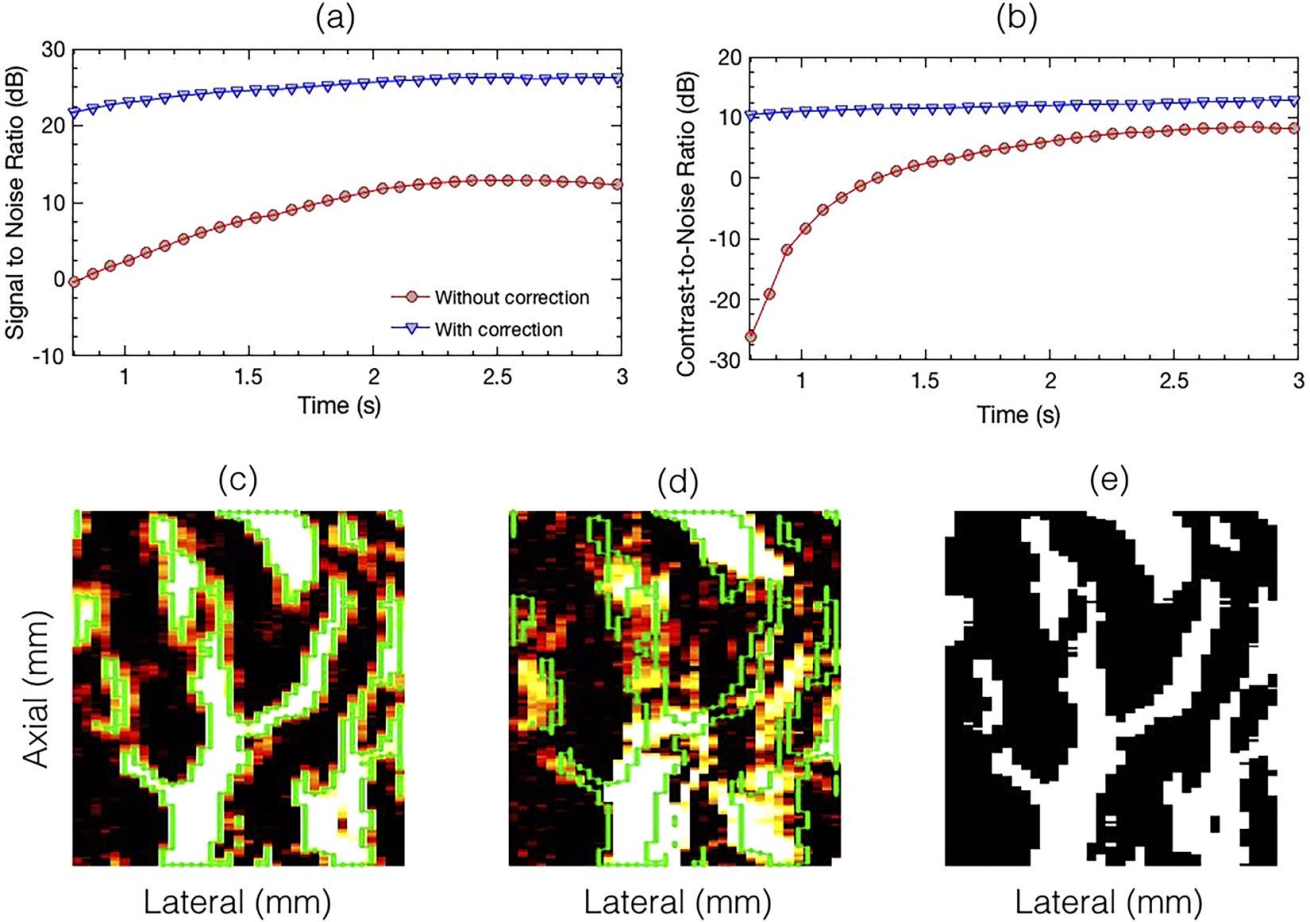
Displays the variation in SNR (**a**) and CNR (**b**) as a function of time. Figures (**c**–**e**) displays the mask generated using an empirical threshold of 20 dB for segmenting the signal and background regions required for calculation of SNR and CNR (Eqns 3 and 4). Figure (**c**) and (**d**) corresponds to PD image obtained with and without motion correction, respectively.

Figure 11 displays SNR (a) and CNR (b) as a function of ensemble length, for PD images computed with (d) and without (c) motion correction, for patient 1. The ROI selected for the quantitative analysis is displayed in (c, d). The binary mask corresponding to the signal and background region, obtained from the motion corrected PD image is displayed in (e). The results show that at all ensemble lengths, both SNR and CNR were relatively higher in the motion corrected PD images. Further, the results displayed that due to lack of coherent integration in the absence of motion correction, a larger number of slow-time Doppler samples were necessary – consistent with observations from Fig. 6.

## Discussion

Spatiotemporal clutter filtering can significantly improve the sensitivity of power Doppler imaging, without using contrast agents^15^. However, in the presence of motion, coherent integration of the clutter filtered Doppler signal can be challenging–especially for small vessel imaging^19^. This limitation could hamper reliable assessment of small vessel blood flow, and limit the scope of PD imaging in the clinic. In this paper, we introduced a first-order rigid body based motion correction technique to address this shortcoming. The efficacy of the proposed technique was demonstrated on thyroid nodules, which incurred large physiological motion due to its proximity to the pulsating carotid artery. The results indicated that the quality of the PD images obtained using the conventional approach (Fig. 1d) were sub-optimal (Figs 4–6). However, motion correction of the Doppler ensemble enabled coherent integration of the back-scattered blood flow signal, which noticeably improved the quality of the PD images (Figs 8 and 9).

The impact of motion on the visualization of small blood vessels in thyroid nodules can be observed in Figs 2 and 3 for patients 1 and 2, respectively. In the absence of motion correction (h–k), the PD images (b,d,f) were noticeably corrupted due to signal distortion, motion blurring and occurrence of shadow vessels, resulting in visibly low signal-to-noise contrast. Further, the incoherency of the Doppler ensemble was evident in the PD intensity profiles (Fig. 6), obtained from two half-ensembles. As a result, the full ensemble PD signal displayed multiple incoherent peaks instead of a single strong peak (Fig. 6(c,f)), thus creating visuals of artificial, low-intensity (shadow) blood vessels (Figs 4 and 5), similar to that illustrated in Fig. 1a–c. However, the distinction between real and artificial vessels was possible in Figs 4 and 5. Specifically, the half-ensemble PD images incurred less motion and thus demonstrated more regular vessel structures that were consistent from (a) to (b), and also with the corresponding motion corrected PD images (d–f). However, in comparison, the corresponding full-ensemble PD image (c) was noticeably degraded in the absence of motion correction. More importantly, motion induced artificial vessels can affect the reliability of the technique for two reasons: (1) it can potentially create a misleading perception of increased vasculature, as observed in the PD images (Figs 3 and 5) of patient 2, and (2) it can considerably reduce the strength of the power Doppler signal, as observed in (Fig. 6a,d) and thus reduce the SNR and CNR of the image. As a consequence of the later, PD images obtained from patient 3 and 5 (Fig. 7) displayed reduced vasculature. These limitations can hamper the scope of this technique in the clinic.

The improvement in image quality upon motion correction was evident for all 10 patients, both qualitatively (Fig. 8) and quantitatively (Fig. 9). The signal amplitude profiles (Fig. 6) revealed large spatial overlap between the half-ensemble signals, which produced a stronger PD peak upon integrating the Doppler signal, as expected in the absence of motion. Consequently, a 1.5–1.7 times gain in signal amplitude was observed upon using motion correction, relative to the uncorrected data (Fig. 6c,f). The time frequency analysis of the Doppler ensemble further elucidated the significance of motion correction in estimation of blood flow spectral characteristics. The pulsating, narrow-band power spectrum associated with blood revealed the spatiotemporal continuity of the Doppler signal at the target location in the motion corrected ensemble, which was otherwise split over adjacent spatial locations in the uncorrected data (Fig. 7). Although, the signal associated with blood flow in displayed examples was observed to be between 250–300 Hz, however this could vary due to aliasing because of the limited frame rate of 568 frames per second for patient 1.

The value of rigid body based motion correction in perfusion imaging has also been emphasized in recent studies involving contrast agent based micro-vasculature imaging^19,20^. The feasibility of the technique was demonstrated on a rat model for renal blood flow imaging. The results obtained from the previous contrast agent based studies and the current study were in good agreement. However, the improvement in visualization of blood flow upon motion correction was more noticeable and pronounced in the absence of contrast agents, primarily due to the relative low intensity of the blood signal which may easily distort and degrade due to motion. Accordingly, the length of the Doppler ensemble assumes an important role in determining the quality of the power Doppler image, since a large and a coherent Doppler ensemble can significantly boost the sensitivity of detecting small vessel blood flow Fig. 11(a,b).

The importance of using ultrafast acquisition for non-contrast agent based Doppler imaging is that it can minimize the overall tissue motion in the Doppler ensemble^15,16^. This enables high spatiotemporal coherence of tissue components, which is important for effective discrimination between tissue and blood, and also for coherent integration of the Doppler signal^15^. However, ultrafast imaging is currently a resource intensive feature, and thus limited to high-end clinical scanners. The technique proposed in this paper can be helpful in improving the performance of SVD based Doppler imaging in mid-range clinical scanners, which may have relatively lower frame-rate due to reduced number of receive channels. In this study, the efficacy of the proposed technique was demonstrated using an Alpinion E-Cube 12R clinical ultrasound scanner, which could acquire data with only on 64 of 128 transducer channels at a time, reducing the imaging frame-rate by half. Further reduction in frame-rate was also due to angular compounding of plane wave acquisitions, which is necessary for improvements in image quality^15–17,35–37^. Additionally, imaging of deeper or larger nodules and its peri-nodular vascularity can further reduce frame-rate and increase the impact of motion. The subsequent loss of spatiotemporal coherence due to low frame-rate can be minimized by using motion correction, as proposed in this study. Another important scope of motion correction for Doppler flow imaging, which is currently our work in progress, is to achieve ultra-long coherent image acquisition to substantially boost the sensitivity of Doppler imaging in visualizing blood flow in micro-vessels (Fig. 11), at relatively low frame-rate. This will allow adherence with the ultrasound imaging safety limits, specifically the thermal index and probe surface temperature rise, which depends on pulse repetition frequency (or frame-rate), and also prevent any potential damage to the probe due to heating.

Motion associated with thyroid nodules were observed to be predominantly in the lateral direction (Fig. 10). This was expected with respect to the anatomical positioning of the thyroid gland with respect to the pulsating carotid artery. Conventionally, tracking of the lateral component of motion using ultrasound can be challenging due to the absence of phase information in the direction orthogonal to beam propagation. However, several researchers have demonstrated that compounded plane wave imaging can reliably estimate lateral displacements associated with carotid artery motion, and optimized its performance^36–40^. In the current study, correction of motion for the individual angular plane wave frames prior to compounding was not performed^36–40^, however, given that the Alpinion US scanner received with 64 channels, motion correction of the plane wave images prior to compounding can be of significance for imaging deeper lesions at low frame rates^41^. Further, the maximum angle for plane wave compounding was 3°; this can lead to lower imaging resolution in the lateral direction and increased artifacts from off-axis bright scatterers. In future studies, this shortcoming will be addressed by increasing the maximum angle and the step size by a factor of 2, without changing the imaging frame-rate. Further, mitigating the issue of bright reflectors is challenging using global SVD based singular value thresholding of clutter. Song *et al*. proposed a block-wise, adaptive framework for local SVD based clutter filtering to address this issue more effectively^35^. Overall, plane wave imaging provides two specific advantages over the conventional Doppler acquisition for visualization of small vessels – (1) Increased frame-rate, (2) increased overall transmission energy by activation of 128 transmit elements compared to 32 or 64 for conventional US imaging. However, it lacks transmit focus, which can affect the sonographic SNR and the penetration depth relative to that achieved using a focused ultrasound beam. Results obtained from rat brain imaging studies demonstrated convincingly that PD images obtained using plane wave imaging had higher SNR compared to that obtained from conventional linear array imaging, due to the use of full aperture^33^.

Further, the pulsation in the axial and lateral displacements estimated using the 2D normalized cross-correlation were observed to be very similar to those reported in carotid artery elastography studies (Fig. 3)^42,43^. The displacements observed across subjects varied, and were dependent on multiple factors such as the size, position and stiffness of the nodule, and its distance from the carotid artery. Accordingly, the pulsation in the displacement estimates were relatively more distinct for patient 2 (Fig. 3), compared to patient 1 (Fig. 2). Additionally, a steady amount of translation motion was observed, possibly due to the slipping of the nodule or the probe, or possible body motion during image acquisition (Figs 2 and 3).

All ultrasound images were acquired across the longitudinal cross-section of the thyroid nodules. The premise on which this choice was based on is that imaging of longitudinal cross-section will incur less out-of-plane motion, due to restricted elevational motion in the presence of trachea in the transverse plane. However, on the contrary, imaging across the transverse plane may be effective in limiting lateral motion, however, at the cost of (1) increased out-of-plane motion along the longitudinal axis – currently measured as lateral motion in the longitudinal plane – that cannot be corrected or accounted for using a 1D probe. (2) Increased lateral strain in the nodule due to restricted motion, which can degrade the quality of the PD image and hinder the scope of motion correction. A detailed *in vivo* study to understand and quantify these tradeoffs will be valuable, however, it was beyond the scope of the current paper, and will undertaken in our future work.

In this study, the patient population lacked positive pathology cases. This was mainly due to two reasons: (1) the recruited subjects were imaged prior to their biopsy, so their pathology was unknown at the time of the study, (2) since majority of the thyroid nodules recommended for biopsy are benign, the chances of studying malignant nodules in a small patient population of 10 subjects is limited. However, we don’t expect the pathology to have any impact on the performance of the proposed technique. Further, the nodules observed in this patient population were shallow – in the range of 2–3.5 mm. In the case of deeper nodules, factors such as increase in attenuation, decrease in the Doppler ensemble size, and higher inter-frame motion due to reduced frame-rate can negatively impact the overall quality of the PD images. Correspondingly, motion correction of the Doppler ensemble obtained from deeper thyroid nodules can be specifically useful in improving the PD image quality. A limitation of our current study is that a global motion correction approach was used in this study, assuming rigid body translation of the thyroid nodule between consecutive frames. This was based on low variance of displacement estimates observed between successive frames in the target thyroid nodules (Figs 2(h,i) and 3(h,i)). In our future work, we will address this limitation by performing motion correction in localized overlapping windows. This will allow motion correction in smaller local kernels that can be reasonably assumed to have uniform displacements. This will also reduce the need for segmentation of the nodule, which otherwise can impact its capability for real-time imaging. Another approach to motion correction would be to use the averaged displacements from the entire frame, however, presence of carotid artery, jugular vein and trachea can impact reliable compensation of motion for small vessel imaging of thyroid nodules.

## Methods

### Data acquisition

The ultrasound in-phase and quadrature (IQ) data for the phantom and *in vivo* experiments were acquired using an Alpinion E-Cube 12R ultrasound scanner (Alpinion Medical Systems Co., Seoul, South Korea), equipped with a L12-3H linear array probe. The plane wave (PW) IQ data was acquired for 7 compounding angles, with a maximum angle of 3° and a step-size of 1° ^17,36,44^. The scanner transmitted and received using 128 and 64 elements, respectively. Accordingly, each angular plane wave transmission was repeated twice and the received data was interleaved for each half of the transducer to emulate a 128 element receive aperture. At transmission frequency of 11.5 MHz, the pulse length of two cycle excitation signal was 67 *μ*m. Subsequently, the received signal was sampled at 40 MHz. The Doppler ensemble was acquired over 3 seconds, and the frame-rate (FR) and pulse repetetion frequency (PRF) varied with the depth and size of the nodule, and is reported in (Table 1). The axial and lateral length of each pixels were 38.5 *μ*m and 200 *μ*m, respectively. The speed of sound was assumed to be 1540 m/s for the calculation of beam-forming delays. Further, the size of the main-lobe of PSF at full-width half-max (FWHM), measured in simulation at 2.5 mm depth on axis, was 70 *μ*m and 177 *μ*m, in the axial and lateral direction, respectively. These calculations were done in simulation to realize a perfect point scatterer, which could otherwise influence the FWHM measurements.

**Table 1:** Patient characteristics and the corresponding *in vivo* imaging parameters.

Patient No.	Gender	Age (yrs)	Side	Depth (cm)	Frame Rate	PRF	Ensemble Size	Pathology
1	Female	73	Right	3	616	8624	1848	Benign
2	Female	58	Left	3	616	8624	1848	Undetermined
3	Female	58	Right	3.5	585	8190	1755	Benign
4	Female	59	Left	2.5	660	9240	1980	Benign
5	Female	44	Left	3	616	8624	1848	Benign
6	Female	55	Left	3	616	8624	1848	Undetermined
7	Female	82	Left	2	702	9828	2106	Undetermined
8	Female	63	Right	2.5	660	9240	1980	Benign
9	Female	47	Right	2.5	660	9240	1980	Benign
10	Female	55	Right	2.5	660	9240	1980	Benign

The imaging frame-rate and PRF depended on the depth of imaging, which was configured based on the size and location of the thyroid nodule. Patients with ‘undetermined’ FNA biopsy outcome were recommended to follow up in 6 months.

### *In vivo* study

The *in vivo* study was conducted to evaluate the feasibility of using motion corrected ultrasound data to improve the visualization of small vessel flow in the thyroid, using a clinical ultrasound scanner. The ultrasound data was obtained from 10 patients with at least one suspicious thyroid nodule, recommended for US-guided FNA biopsy. The study was performed in accordance with the relevant guidelines and regulations approved by the Mayo Clinic institutional review board and an approved, informed consent in written was obtained from the patient prior to their participation. Although, all recruited subjects were females, we don’t expect any impact of gender on the hypothesis or on the outcome of this study. The ultrasound data was acquired by an experienced sonographer, across the longitudinal cross-section of the right thyroid gland, prior to the scheduled biopsy of the nodule. Further, the subject was asked to hold her breath for the 3 seconds duration of the scan to minimize any motion due to breathing. Additional details about the subjects and imaging parameters are listed in Table 1.

### Displacement tracking

The axial and lateral displacements associated with the *in vivo* ultrasound data were estimated using a 2D normalized cross-correlation based echo tracking algorithm. The displacement estimates were obtained prior to clutter filtering, although motion correction was applied on clutter-filtered data. The ultrasound RF images were interpolated in the axial and lateral direction by a factor of 3 and 10, respectively, to increase the spatial density of correlation functions^45–47^. Subsequently, the ultrasound RF images were tracked using a 2D kernel (0.2 mm × 0.8 mm), which overlapped by 90% in both coordinates. The Cartesian displacement estimates between each consecutive pair of RF images were calculated as described in^48^, using peak of cross-correlation. Specifically, the 2D kernel based sliding reference window obtained from the first ultrasound RF image was cross-correlated with the corresponding search region in the subsequent image. The location of highest cross-correlation coefficient estimated the corresponding displaced location of the reference window in the subsequent RF image. Additionally, a 2D spline interpolator was used to determine the location of the peak cross-correlation co-efficient to sub-pixel accuracy. Further, The 2D cross-correlation based displacement tracking was performed on a Titan XP GPU (Nvidia Corp., CA, US). The estimated axial and lateral displacements maps were transformed from Eulerian to Lagrangian co-ordinates, to correspond with the first frame of the Doppler ensemble.

### SVD based Spatio-temporal Clutter Filtering

The original and the motion corrected ultrasound data was clutter filtered using the singular value decomposition of the spatiotemporal Casaroti matrix^15^.

1 $${S}_{blood}=S(x,z,t)-\sum _{r=1}^{r=th}\,{U}_{r}{\lambda }_{r}{V}_{r}^{\ast }$$

where the matrices *S* and *S*_*blood*_ represent pre- and post-clutter filtered Doppler ensemble. The matrices *U*, *V* consist of left and right singular orthonormal vectors, respectively. The corresponding singular values and their order are denoted by *λ*_*r*_ and *r*, respectively, and * represents conjugate transpose. A global SV threshold was chosen to separate tissue clutter from blood signal, based on the decay of the double derivative of the SV orders (i.e. when the double derivative approached zero).

### Motion correction

Subsequently, motion correction of the Doppler ensemble was performed to re-register each ultrasound frame with that of the first frame, by globally shifting the rows and columns by the estimated displacements. Specifically, the mean axial and lateral displacements obtained from each frame, averaged over the demarcated tumor nodule, were used to correct for motion. Further, the real and imaginary components of the IQ data were tracked and motion corrected independently, prior to the power Doppler estimation.

### Power DOPPLER Imaging

The final power Doppler signal was computed from the motion corrected data:

2 $$PD(x,z)=\sum _{t=1}^{{N}_{t}}\,|{S}_{blood}(x,z,t{)|}^{2}$$

where *N*_*t*_ denotes the ensemble length. The background separation in the PD image was achieved by using a top-hat morphological filter, with a disc-shaped structuring element of radius 10 pixels^49^.

### Data analysis

Quantitative assessment of the imaging performance was performed by estimating the signal to noise ratio (SNR) and contrast to noise ratio (CNR) of the power Doppler images:

3 $$SNR=20\ast lo{g}_{10}(\frac{{\mu }_{v}}{{\mu }_{bg}})$$

4 $$CNR=20\ast lo{g}_{10}(\frac{|{\mu }_{v}-{\mu }_{bg}|}{\sqrt{{\sigma }_{v}^{2}+{\sigma }_{bg}^{2}}})$$

where *μ* and *σ* denote the mean and the standard deviation of the signal, respectively. Further, the subscripts *v* and *bg* correspond to signal obtained from the vessel and background regions, respectively. A constant offset of 10 dB was added to all estimated CNR values to display a positive estimate, specifically, in the absence of motion correction.

The CNR and SNR metrics were computed in each of the rectangular regions of interest (ROI) identified in Fig. 8. These rectangular ROIs comprised both vessel and background pixels. A threshold of 20 dB was empirically selected to separate the vessels signals from the background. This generated a mask for vessel and background pixels that was subsequently used for CNR and SNR computation. A representative example has been displayed in Fig. 11. Subplot (c) demonstrates the mask generated from the motion corrected power Doppler image with a threshold of 20 dB. The white and black pixels represent the vessel and background signals, respectively. Based on the evidence demonstrated in Figs 4–7, the mask was generated from the motion corrected PD image.

## Conclusion

In this study, we demonstarted that motion from the carotid artery can noticeably impact power Doppler imaging of small vessels in thyroid. To address this issue, we proposed motion correction of the clutter filtered ultrasound data prior to power Doppler computation. The efficacy of the proposed technique was demonstrated using compounded plane wave imaging implemented on a commercial ultrasound scanner, on ten human subjects with thyroid nodules suspicious of malignancy. The results demonstrated that the proposed technique considerably improved the visualization and detection of small vessel blood flow in thyroid. This technique is particularly valuable in non-contrast agent based power Doppler imaging, since the the low intensity signal contribution can be easily corrupted due to motion. These preliminary results suggests that proposed technique performs sufficiently well to warrant further development and more *in vivo* validation.
